# Integrating high-performing electrochemical transducers in lateral flow assay

**DOI:** 10.1007/s00216-021-03301-y

**Published:** 2021-04-28

**Authors:** Antonia Perju, Nongnoot Wongkaew

**Affiliations:** Institute of Analytical Chemistry, Chemo- and Biosensors, University of Regensburg, 93053 Regensburg, Germany

**Keywords:** Lateral flow assay, Electrochemical detection, Point-of-care devices, Electrochemical transducers, Nanofibers

## Abstract

Lateral flow assays (LFAs) are the best-performing and best-known point-of-care tests worldwide. Over the last decade, they have experienced an increasing interest by researchers towards improving their analytical performance while maintaining their robust assay platform. Commercially, visual and optical detection strategies dominate, but it is especially the research on integrating electrochemical (EC) approaches that may have a chance to significantly improve an LFA’s performance that is needed in order to detect analytes reliably at lower concentrations than currently possible. In fact, EC-LFAs offer advantages in terms of quantitative determination, low-cost, high sensitivity, and even simple, label-free strategies. Here, the various configurations of EC-LFAs published are summarized and critically evaluated. In short, most of them rely on applying conventional transducers, e.g., screen-printed electrode, to ensure reliability of the assay, and additional advances are afforded by the beneficial features of nanomaterials. It is predicted that these will be further implemented in EC-LFAs as high-performance transducers. Considering the low cost of point-of-care devices, it becomes even more important to also identify strategies that efficiently integrate nanomaterials into EC-LFAs in a high-throughput manner while maintaining their favorable analytical performance.

**Figure:**
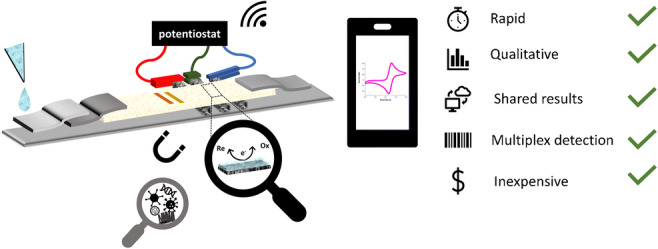
Graphical abstract

Graphical abstract

## Introduction

**Fig. 1:**
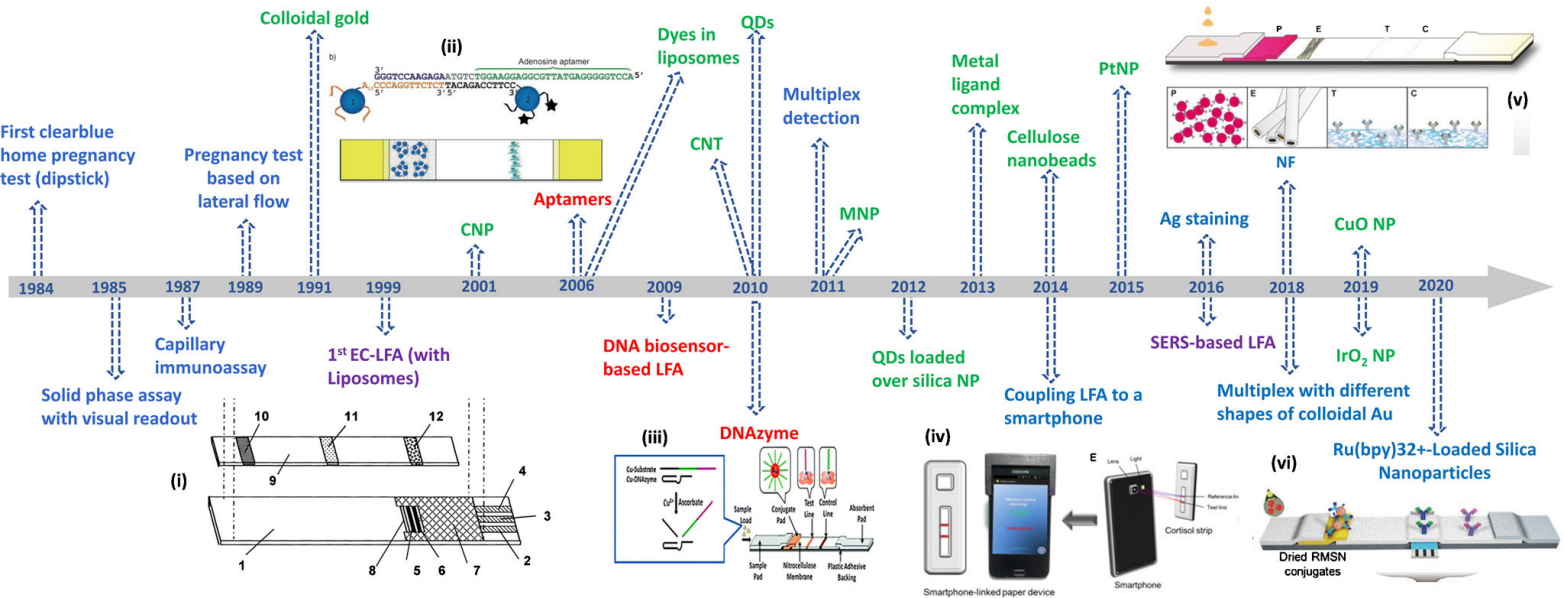
Timeline of the exemplary developments of LFA starting in 1984 with the first clearblue home pregnancy test (clearblue.com), which continued with two important patents: solid-phase assay with visual readout [4] and capillary immunoassay [5], then the first pregnancy test based on a lateral flow test (clearblue.com), leading to the first patent on LFA using colloidal gold [6]. The development continued with the first EC-LFA and first use of liposomes [7], followed by first use of carbon nanoparticles (CNP) [8], then liposomes entrapping dyes [9] and use of aptamers in LFA [10]. Further research led to DNA hybridization detection-based LFA using enzyme as a tracer [11], implementation of DNAzyme for metal ion detection [12], use of CNT [13], QDs [14], and MNP [15], achieving multiplex detection [16]. Further improvements focused on tracer strategies, like metal ligand complexes [17], cellulose nanobeads [18], and the coupling to a smartphone [19]. In the past few years, the research has been focused on further developing labels like Pt-nanoparticles (PtNP) [20], the use of Ag staining for amplification [21], combining LFA with SERS [22], implementing nanofibers (NF) [23], using different shapes of colloidal gold [24], using different nanoparticles (NP) like IrO_2_ [25] or CuO [26], and integrating electrodes for electrochemiluminescence (ECL) [27]. The texts shown in blue, green, purple, and red represent the development focusing on assay format, signal tracer, detection technique, and recognition element, respectively. Figures reprinted from (i) [7], (iv) [19], (v) [23] with permission from Elsevier. Figures reprinted from (ii) [10] and (vi) [27] with permission from John Wiley and Sons. Figure (iii) reprinted from [12] with permission from Royal Society of Chemistry

Lateral flow assays (LFAs) are the most well-known point-of-care (POC) devices that enable rapid detection of relevant bio- or chemical markers in a simple and low-cost manner by non-specialized users. Additional advantages of the LFA include the facile fabrication process and the inexpensive material used in the device preparation. Only a small volume of sample is needed, typically in the microliter range, and the sample does not require special pre-treatment, allowing a feasible sample-to-answer process. LFA usage was primarily aimed towards medical diagnostics as home-based testing devices or used in clinical laboratories, but their applicability has later extended to agriculture, food safety, and environmental monitoring [1]. It dates back to 1969 when Margaret Crane first patented a diagnostic test device concept applicable for home testing of pregnancy [2]. This seminal work led to further developments in various versions prior to the commercial home pregnancy test based on LFAs as can be seen in Fig. 1. Lateral flow strips (LFSs) have found rapid successful commercialization due to the following attractive characteristics—Affordable, Sensitive, Specific, User-friendly, Robust & Rapid, Equipment-free and Deliverable (ASSURED)—that meet the demands of a POC device. As can be seen in Fig. 1 the principle has actually emerged a long time ago and set the foundation for a number of interesting subsequent developments. Nowadays, utilizing gold nanoparticles (AuNPs) as a signal tracer remains a gold standard in LFA that allows detection of the color change by naked eyes. Despite the simplicity and rapid signal generation of AuNPs, many attempts have been made towards improving detection sensitivity as the target analytes found in body fluids are normally present at low concentrations that may lead to false-negative prediction or detection [3]. More importantly, LFAs that can quantify analyte concentrations will be desirable to serve in a wide range of applications.

In order to improve the sensitivity of LFAs, many attempts have targeted detection techniques, using new transducer materials and enhancing signal through effective amplification strategies and the use of nanomaterials. Another important aspect is to develop LFAs with a quantitative readout which plays an important role in medicine, agriculture, food safety, and environmental monitoring. Especially in the medical area, the specific pathogen concentrations or level of clinical relevant markers [28] will determine the proper treatment. Qualitative LFAs may be sufficient for some applications such as the detection of human chorionic gonadotropin (hCG) in a pregnancy test. However, for some diseases, e.g., diabetes, knowing the concentration of an analyte is crucial to make further treatment decisions. Other analytes whose concentration levels are highly relevant are metabolites, hormones, and blood-borne chemicals [29]. The metabolites which are targeted by most POC devices are cholesterol, triglycerides, creatinine, lactate, ammonia, and urea. For example, creatinine is used for the assessment of renal function; lactate for tissue perfusion and presence of ischemia or hypoxia; and urea and ammonia levels for diagnosing renal dysfunction, liver disease, or asthma. Moreover, the focuses of POC diagnostics lie also on metabolites of regulated or illegal drugs, such as cocaine, opiates, and cannabis [29].

Strategies using electrochemical detection offer attractive features such as high sensitivity, selectivity, low instrumentation cost, and inherent miniaturization ability with improved analytical performance. So far the developments of LFA-based electrochemical detection have utilized disposable transducers such as screen-printed electrodes (SPE), disposable gold electrodes built from recordable compact discs or indium tin oxide (ITO) [30], which are well suited for single use in an LFA. Moreover, the development of nanomaterials offers the possibility to enhance the performance of the transducers, e.g., by increasing their conductivity, enabling electrocatalytic effect, enhancing electron transfer rate and increasing the surface area of the electrodes [30]. The nanomaterials with various dimensions (0D-3D) have been successfully implemented to miniaturized analytical systems as shown in many studies [31]. Electrochemical transducers of 3D-architecture are more attractive for use in LFAs than those of other dimensions as sample liquid can pass through the transducer, promoting efficient interactions between analyte and transducer surface. In addition, the increased electroactive surface area could possibly extend the dynamic range of the sensor.

With LFAs being widely studied, there are many reviews available in the past and present. This indicates that the developments of LFAs are still very active and in focus by many researchers around the world. An interesting review focusing on recent developments towards quantitative LFAs highlighted that the majority of devices reaching commercialization are based either on colorimetry or on fluorimetry. They address a number of advantages for developing quantitative LFAs [28]. A more recent review focused on detection strategies available for LFAs has also been published by Nguyen et al. [32]. Moreover, there have been many reviews [1, 3, 33–37] on LFAs with foci on different aspects of LFA, for example, directed flow on LFAs in the form of microfluidic paper-based analytical devices [34], or on nanoparticles used in LFAs [38]. To the best of our knowledge, despite their many beneficial features mentioned above, less attention has been paid to LFAs operating with electrochemical detection which could be likely because the integration of electrochemical transducers to LFSs requires complicated fabrication methods. However, non-obvious fascinating analytical performance is possible in this format, especially in comparison to optically based detection techniques. Furthermore, with ongoing progresses in material science with respect to facile fabrications strategies available, it could be seen in the future that LFA-based electrochemical detection is one of the most interesting areas to put research efforts on. Therefore, in this trend article, we want to emphasize the benefits of integrating electrochemical transducers onto LFSs for achieving quantitative results, by merging the advantages of a POC device with those of electrochemical detection. At the beginning, we describe recent developments of recognition strategies and signal tracers. Then, the common techniques used in LFAs are described. We further take into consideration all the advances that have been made in the last years to fabricate electrochemical LFAs and we propose how recently developed transducers could be integrated into LFS. At last, a future perspective is intended to highlight the possibilities and advantages of electrochemical LFA. The overall purpose of this trend article is to propose beneficial integration strategies for high-performing electrochemical transducers onto LFSs.

## Principle of LFAs

**Fig. 2:**
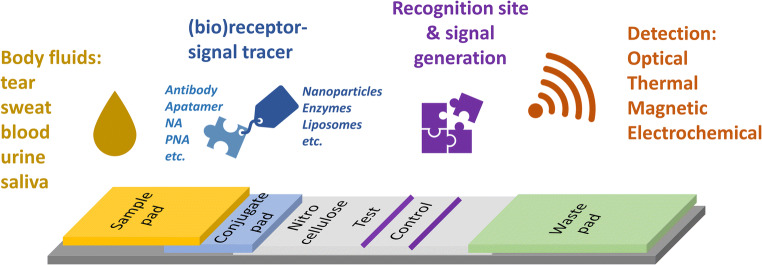
The components of an LFA

The LFA requires low sample volumes to apply on a porous membrane, flowing further along the entire strip that consists of different connected zones with specific functions (Fig. 2). As illustrated in Fig. 2, a sample pad is the zone located at the beginning of the strip where fluid sample is applied. This zone typically contains pre-dried buffering salts and surfactants to pre-treat the sample, if necessary. The reagents become rehydrated when the sample passes through. The sample solution from the sample pad is further delivered to a conjugated pad positioned underneath an overlapping nitrocellulose (NC) membrane. Here, (bio)-recognition elements anchored with signal labeling are stored at the conjugated pad. The target analyte bound to the conjugated receptors further travels along the NC membrane where a detection zone is located. In general, the detection zone consists of two lines namely test and control lines. In the test line, a recognition element complementary to the analyte is immobilized and responsible for capturing the analyte complex. In the control line, the recognition elements anchored with signal labeling will be trapped to confirm a proper functioning of the strip [35]. The last part of the LFS is called a waste pad which is overlapping with the NC membrane. This part is responsible for promoting the flow and preventing the back flow of the sample solution. It is important to note that all pads must slightly overlap when assembled on the backing substrate, to ensure a proper fluidic contact between zones that allows the solution to be driven by the capillary forces smoothly till the end of the strip.

In general, the LFSs applied to immunoassay can be performed into two formats, namely direct and competitive assay. The direct assay is typically performed via a sandwich format, where the antigen (analyte) binds first to the specific primary antibody present in the conjugate pad and is then captured by the secondary antibody on the test line. Therefore, the presence of signal in the test line indicates a positive result. This format is suitable for analytes of relatively large size which have multiple binding sites, e.g., p24 antigen used for HIV detection and hCG used in the home-based pregnancy test. In a competitive assay, antigen (analyte) is immobilized in the test zone; therefore, the labeled receptor can be captured, generating the signal when the target analyte is absent. On the other hand, the presence of analyte in a sample will hinder the binding event of labeled receptor, thus hampering the signal generation in the test line. Therefore, a lack of signal or reduction in signal intensity at the test line indicates a positive result [35]. Similar to the direct assay format, antibodies specific to labeled receptor is immobilized at the control line to confirm proper functionality of the system. The competitive assay is more flexible as it can be applied to any analyte of interest without the restrictions of available binding sites and sizes. The construction concept shown in Fig. 2 and the two assay formats remain the standard configuration for LFA development nowadays.

## Current developments of LFAs

This section aims to provide readers with a broad overview on what has been done so far to improve performance of LFAs, especially in terms of selectivity and sensitivity, for non-electrochemical readings. The developments of novel recognition probes can be definitely implemented to electrochemical LFAs to promote high selectivity and stability. The developments of signal tracers for other detection techniques can be promising for electrochemical readings as some of them are inherently electroactive.

### Recognition strategies

Recognition elements are a crucial part in LFAs as they determine specific reactivity to target analytes. Ideally, real sample solution is expected to be directly applied to LFS without any pre-treatments. Therefore, recognition elements should be highly stable in the sample solution of interest. Binding between biorecognition elements and analytes should be fast as no pre-incubation is present in ideal LFAs. The most common biorecognition elements present at a conjugate pad and test zone (test and control line) are antibodies (Abs) as they are very well-established, especially in terms of immunoreactivity, production, and commercial availability. However, they still suffer from drawbacks, such as long production time, poor stability, strict storage conditions required, and batch-to-batch variation [39]. As a solution to these problems, aptamers have nowadays gained more attention as counterparts to Abs in LFAs, as they are stable, are simpler to use, and have lower costs than Abs [40]. Aptamers, also known as chemical Abs, are made from nucleic acid (NA) in which their complex folding into 2D or 3D structure allows them to bind selectively to corresponding analytes with high affinity [41]. The change of their conformation upon binding to analytes is also highly suitable for label-free assays, in particular for small molecule detection [42]. Aptamers have been successfully implemented to LFAs with good detection sensitivity and specificity not only for clean samples such as sweat [40] and saliva [43] but also for challenging samples such as the avian influenza H5N2 virus particle spiked in solution containing duck’s feces [44]. With their attractive features and progresses in terms of production and a vast variety of available NA sequences for specific analytes, aptamers possess a great potential to substitute the use of Abs in LFA in the coming future.

Oligonucleotide sequences have been commonly used for detection of their complementary target sequences. However, most detection techniques in LFA are not sensitive enough to enable direct detection after NA isolation and purification. Therefore, NA targets are commonly pre-amplified, then termed DNA amplicons, prior to the detection on LFA [45]. The amplification can be performed either off- or on LFS [46]. The resultant double-stranded DNA amplicons (such as from loop-mediated isothermal amplification (LAMP)) typically require denaturation into single-stranded DNA prior to introducing into LFS for the hybridization assay [47]. However, Jauset-Rubio et al. demonstrated the possibility to detect double-stranded DNA amplified by recombinase polymerase amplification (RPA) [48]. Herein, the use of tailed primers in RPA provides DNA amplicons with duplex flanked by two single-stranded DNA tails. Therefore, direct hybridization to immobilized capture probes and labeled reporter probes are feasible.

Molecular beacons are short single-stranded DNA sequences that can form a hair pin loop structure with a 15–30-base pair-long region complementary to the target sequence and 4–6 base pairs that form the stem [36]. Molecular beacons are suitable for detection of short NA sequences without the need of a second reporter probe, thus lowering the assay cost. A labeling molecule is anchored at the end of the molecular beacon probe. Upon binding to complementary target NA, the stretching of the probe alters the signal transduction property of the labeling molecule, thus enabling detection of the binding event.

Peptide nucleic acids (PNAs) are DNA mimics with neutral polyamide backbone and have gained a lot of attention as alternatives to DNA probes, in particular for short NA sequences such as micro-RNAs (20–22 mers) that are unlikely to be effectively amplified [49]. Here, the polyamide backbone in PNA probes enables the formation of a PNA-RNA duplex with higher binding strength than those of RNA-RNA or DNA-RNA. The neutral backbone of PNA contributes to the highly strong binding of NA as electrostatic repulsion between the two hybridized strands is eliminated. In addition, background signal in electrochemical detection is considerably lower due to the uncharged backbone. Thus, monitoring the hybridization event of PNA with DNA or RNA based on electrochemical label-free techniques is feasible as demonstrated in previous studies where charge of the probe plays an important role for signal generation [48, 50, 51]. LFA combined with PNA probes has been successfully implemented to determine micro-RNA-150 in plasma samples for preterm birth screening test [52].

Nucleic acids with catalytic cleavage reactions, e.g., DNAzymes, are an interesting recognition element for metal ion detection in LFAs [53]. In principle, the DNAzyme sensor for metal ion detection consists of substrate and enzyme strands [54]. Here, metal ions serve as a cofactor of the DNAzyme complex and induce cleavage of the substrate strand. The cleaved substrate strand can then be detected by various techniques [55].

Moreover, peptides that consist of short amino acid chains can be an attractive candidate for biorecognition elements [56]. Specific peptide sequences can recognize various kinds of analyte molecules including proteins, metal ions, and NAs. Some peptide sequences can act as a substrate that can be cleaved by proteases [57]. Therefore, peptides can be used as a recognition probe for determining protease activity which is an important biomarker in many diseases; for example, matrix metalloproteinase-9 (MMP-9) is a well-known protease for cancer diagnosis as it plays a crucial role in the cell invasion and tumor metastasis [58].

### Signal tracers and non-electrochemical readings

Recent developments have proposed the use of advanced labels based on naked-eye detection, fluorescent or chemiluminescent reading, and surface-enhanced Raman spectroscopy (SERS). Alternative to these optical readings, measurements of physical properties such as magnetic strength and generated heat have been exploited in LFAs but are not as popular as the optical methods [59]. In this section, we therefore focus mainly on optically based detection strategies.

Naked-eye detection remains the most popular choice over the other options due to instrument-free readout. Gold nanoparticles (AuNPs) are the most popular signal tracer in LFAs because they deliver a strong signal that is visible to the naked eye and moreover are easy to synthesize, stable, and uncomplicated in handling [38]. However, to improve their detection sensitivity and to develop the LFA strategy towards a semi- or fully quantitative detection, many research efforts have been focused on other nanomaterial tracers. For example, to increase signal intensity via naked-eye detection, nanoparticles that allow more intense color than AuNPs or high contrast signal over the white background of the LFA membrane have been proposed. These included nanoparticles based on Prussian blue [60], carbon nanoparticles (CNPs) [61], and magnetic nanoparticles (MNPs) [62]. Enhancement of colorimetric signal of AuNPs before or after capturing at the detection zone is an alternative strategy [63]. This could be carried out by (i) increasing the density of AuNP tracers, e.g., using AuNP aggregates; (ii) increasing particle size through silver or gold deposition; and (iii) decorating AuNPs with catalysts, e.g., enzyme or metal, that enables greater signal generation than obtained from AuNPs alone.

Apart from inorganic nanomaterials, enzymes have also been used as a signal tracer for optical detection in LFAs [64]. Here, substrate reagent is needed to generate detectable products which introduces one more step in the detection. Horseradish peroxidase (HRP) is the most common enzyme applied to LFAs. Its chromogenic substrate, 3,3′,5,5′-tetramethylbenzidine (TMB), is being oxidized by HRP in in presence of H_2_O_2_, resulting in a product that is visualized on the LFSs [64]. Apart from detecting colored product, a chemiluminescent (CL) signal can be obtained from the catalytic reaction of luminol with H_2_O_2_ via HRP that can be detected by a charged-coupled device (CCD) camera or other analyzer system [65, 66]. The high sensitivity and high signal-to-noise ratio using simple optical readout systems make CL more attractive than other optical readout methods.

The signal enhancement of the LFAs based on optical detection has been also achieved by using fluorescent dyes instead of the metallic NP, resulting in a higher signal-to-noise ratio, which increases the sensitivities and thus lowers the detection limit [32]. The fluorescent tracers such as quantum dots (QDs) are also very popular to implement in LFAs by taking advantage of good sensitivity and high stability of generated signals. QDs can provide a strong photoluminescent signal upon excitation by UV light. The signals can be detected at long wavelengths in which the emission peaks are dependent on size and composition of QDs. Therefore, QDs are suitable for multi-analyte detection in LFA [67, 68]. Alternative to QDs, upconverting nanoparticles (UCNPs) have become more attractive as the excitation wavelength in the near IR region can reduce the tendency of auto-fluorescence of the membrane, unlike when using UV light, and produce a strong signal in the visible region which is more sensitive than that provided by QDs.

Loading the signal tracers into micro- or nanocarriers, e.g., liposomes [7], polystyrene beads [69], MNPs, and cellulose nanobeads [18] has shown to be an effective strategy to dramatically improve detection sensitivity of LFAs for various kinds of signal tracers mentioned above.

Even though the developed detection strategies mentioned above have shown desirable performance when applied to LFAs, the difficulty in commercialization remains a major obstacle as either LFA fabrication, or the necessary instrumentation for readout is expensive. Even so, the ones that are available on the market only offer mostly qualitative or semi-quantitative detection. As mentioned at the beginning, this is sufficient for some diagnostics, but there are a wide variety of analytes that need to be quantitatively determined, as their concentration greatly affects the decision taken for subsequent actions. The visual colorimetric detection often suffers from poor sensitivity and lack of reproducibility. Moreover, subjective visual comparisons of color may still be suitable for qualitative results, but reliable quantitative measurements require objective data interpretation [34]. Even for the fabrication of new nanoparticles, which can offer a great improvement of the sensitivity, the surface properties have to be carefully controlled, to allow an efficient bioreceptor conjugation, low non-specific binding, and smooth migration across the membrane [63]. Although, technologies have evolved and higher sensitivity is possible to achieve, in most cases, simplicity is compromised by involving multiple steps or even complicated procedures. For example, for quantification of visual signals, optical or fluorescent readers or a camera could be employed. However, these require a complicated software for measuring and analyzing the intensity of the signal. Even if there is an enormous advantage to use the smartphone for both imaging and analyzing, the data has to be normalized to the specific characteristics of the camera used for data acquisition and a consistent lighting has to be ensured, in order to yield reliable results.

## Electrochemical lateral flow assay (EC-LFA)

Electrochemical transduction is attractive to be integrated into LFAs due to its simplicity, high sensitivity, fast signal generation, and cost-effectiveness. Moreover, EC-LFAs benefit from large detection range, high reproducibility, and possibility for real-time measurements. The miniaturization together with high analytical performance can be easily achieved not only in the fabrication of the electrodes but also for the necessary instrumentation, all at a relatively low cost. The advantages of micro- or even nano-sized electrodes promote the use of very small sample volumes and improve the sensing performance, unlike optically based detection where miniaturized system can lead to poorer sensitivity. Another very important characteristic of electrochemical detection is its low level of interferences from complex sample matrices, such as in clinical or food samples, which is a great advantage over the optical detection techniques [70]. Additionally, the development and continuous improvement of the screen-printing technique and ink material formulation facilitate their facile integration into electrochemical biosensors. One of the most desirable characteristics is their cost-effectiveness and mass production possibilities of such systems [32]. The materials used for this technique are also disposable and simple and have low power requirements, quick response, high sensitivity, and the ability to be operated at room temperature [71]. Moreover, the electrochemical performance can be further improved through developing signal tracer in similar manners achievable for naked-eye detection, fluorescent or chemiluminescent readings, i.e., incorporating a large number of signal tracer in a single label. Another interesting advantage of electrochemical detection is the feasibility of label-free detection.

### Modes of signal generation in EC-LFA

The most popular techniques for signal generation in EC-LFAs are voltammetry, amperometry, and impedimetry as can be seen in Table 1. For voltammetry, the current is measured at a potential varying over time, while amperometric current is measured at a constant potential. Voltammetry- and amperometry-based techniques typically require a redox indicator to detect a signal. A well-known and mostly used method for investigating electrochemical reaction at an electrode is cyclic voltammetry (CV). The potential is applied as a triangular waveform, generating current vs potential in a cyclic wave form. Normal pulse voltammetry (NPV), differential pulse voltammetry (DPV), and square wave voltammetry (SWV) are voltammetric methods in which the potential follows more complicated patterns. Alternative to DC potential, electrochemical impedance spectroscopy (EIS), which measures the impedance in dependence of the frequency of the applied AC potential, has been applied to LFA [72]. The electrochemical techniques can be label-based (enzyme, nanoparticles, etc.) or label-free platform. As shown in Table 1, EC-LFAs utilizing SWV [73–78], chronoamperometry (CA) [79–81, 88], chronocoulometry (CC) [82], EIS [72, 83, 89, 90], stripping voltammetry [76, 91], and CV [84, 85] have been demonstrated with low detection limits and wide linear ranges. SWV is the most advanced form of voltammetry, which uses a staircase potential function in combination with potential pulses of constant amplitude [92] to diminish the charging current, present in CV, also enabling a fast measurement and high sensitivity. A simple technique based on CA with good signal-to-noise ratio, still enabling sensitive detection and real-time measurement, is also popular. Stripping voltammetry is considered a very sensitive technique for quantitative determination of small molecules. A pre-deposition step prior to detection allows the intimate contact between the analyte of interest and electrode surface. In LFAs, metallic or ionic signal tracers, e.g., QD containing cadmium [91] and bismuth ions [76] have been used. The acid-induced release of cations from the nanoparticles is initially performed prior to electrodeposition onto electrodes. Multiplex detection is feasible when various signal tracers are used.

**Table 1:** LFA-based electrochemical detection with their analytical performance

Analytes	Electrode type	Method	Signal tracer	Electrode assemble strategy	LOD	Linear range	Reference
Clenbuterol	PANI@GO/ITO	EIS	–	Strip between the electrodes	0.12 ppb	0.12–58 ppb	[72]
Organophosphorus pesticide	SPE	SWV	Acetylcholinesterase	Test zone cut with cutter embedded in plastic case	0.02 nM	0.05–10 nM	[73]
Alpha-fetoprotein	SPE	SWV	HRP	Electrode under test zone	0.5 ng/mL	1–100 ng/mL	[74]
PSA	SPE	SWV	CdSe@ZnS QD	Electrode under test zone	0.02 ng/mL	0.05–4 ng/mL	[75]
hCG	SPE	SWASV	Bi^3+^	Electrode under test zone	1 mIU	0–70 mIU	[76]
Lead and cadmium	SPCE	SWV	–	Electrode over the test zone	7 and 11 ppb	10 to 100 ppb	[77]
Trichloropyridinol	SPCE	SWV	HRP	Test zone cut with cutter embedded in plastic case	0.1 ng/mL	0.1–100 ng/mL	[78]
8-OHdG	CNT paper	CA	–	Electrode over the test zone	3.11 ng/mL	0–150 ng/mL	[79]
Myeloperoxidase	SPE	CA	MBs (separation) and HRP	SPE printed on the membrane	0.18 ng/mL	0.25–8 ng/mL	[80]
Testosterone	Gold electrodes made by lithography	CA	HRP	Test zone cut	1 ng/mL	1–625 ng/mL	[81]
Troponin I	ITO	CC	Gal and An-GP	Electrode over the test zone	0.1 pg/mL	0.1 pg/mL–100 ng/mL	[82]
PSA	Interdigitated-SCPE	Capacitance	Urease	Electrode over the test zone	1 ng/mL	0–30 ng/mL	[83]
Cardiac troponin T	SPE	CV	HRP	Electrode under test zone	0.15 ng/ml	0–700 ng/mL	[84]
Dengue NS1 protein	SPGE	CV	TEMPO-tagged AuNP	Electrode under test zone	50 ng/mL	0–1000 ng/mL	[85]
8-OHdG and PSA	–	Glucose meter	Invertase	Test and control zone cut	0.23 and 1.26 ng/mL	0.1–100 and 1–100 ng/mL	[86]
Hg(II) ions	SPE	SWV	HRP	Test zone cut	30 pg/mL	0.1–200 ng/mL	[87]

*MBs* magnetic beads, *Gal* galactosidase, *AN-GP* 4-amino-1-naphthyl β-d-galactopyranoside, *AN* 4-amino-1-naphthol, *PANI@GO/ITO* polyaniline@graphene oxide/indium tin oxide, *8-OHdG* 8-hydroxy-20-deoxyguanosine, *PSA* prostate-specific antigen, *SWASV* square wave anodic stripping voltammetry

Enzyme-generated electroactive products have been widely studied [73, 74, 78, 81–84, 86, 88] to increase the sensitivity and selectivity of the system. However, the immobilization and stabilization of the enzyme can be complicated, which could also increase the cost of the sensor and affect the overall stability of the system. Moreover, in case the enzyme reaction takes place at the test zone where the electrode and capture probes are located, fast reaction and efficient electron transfer between the enzyme and the electrode is required. However, it is more favorable to place electrodes downstream after the capturing zone.

In contrast to other techniques, electrochemical detection in LFAs can be performed without tracer. For direct assay formats, after analyte has bound to the biorecognition element immobilized on the electrode, an increase in impedance at the electrode electrolyte interface can be monitored. For the competitive format, on the other hand, the impedance is theoretically decreased when analyte is present [72]. In addition to EIS, direct oxidation or reduction of electroactive analyte after binding to biorecognition element immobilized on an electrode can be monitored by CA [79]. The label-free LFAs could be advantageous due to the reduction of assay cost. However, it should be noted that electrode materials must be very sensitive and facilitate efficient electron transfer in order to detect small changes and enable electrochemical reactions, respectively. Therefore, microelectrodes or nanomaterials have been used for this purpose.

## Integrating electrochemical transducers into LFAs

The concept of an LFA coupled with an electrochemical transducer is highly promising as discussed in the previous section. However, fabrication and integration of electrochemical transducers into LFAs are crucial to their successful translation into commercial products. In this section, we aim to give an overview on how electrochemical transducers can be fabricated and integrated into LFAs and discuss the pros and cons of each method.

### External electrodes

**Fig. 3:**
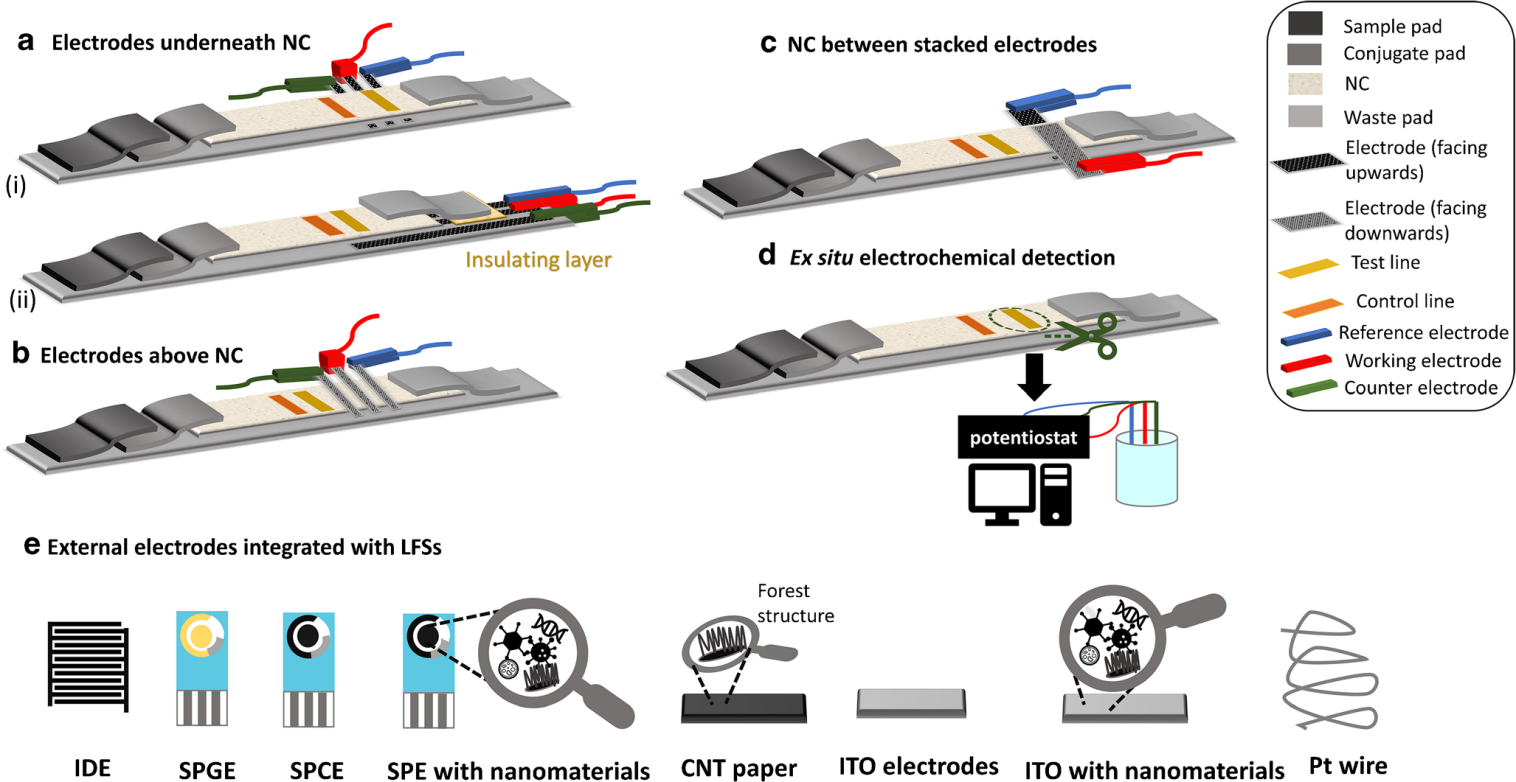
Scheme of existing principles to integrate electrodes into LFS: **A** electrodes underneath NC with the different electrode orientations (i and ii), **B** electrodes placed above the NC with the electroactive surface facing downwards, **C** NC membrane positioned in between the stacked electrodes in a two-electrode configuration, **D** cutting of the test zone and performing electrochemical measurement off-strip, and **E** types of electrodes and modifications which were demonstrated in developing EC-LFA

Screen-printed electrodes (SPEs) are well-established disposable transducers that have been widely combined with LFAs (see also Table 1). Although SPEs have been already employed in LFS for more than two decades [7, 93], the principle in their fabrication technology has basically remained the same. Different integration strategies that have been employed in the past years are summarized in Fig. 3. The external electrode can be mounted either underneath (Fig. 3A) or above (Fig. 3B) the NC membrane at or after the test zone. For the detection of prostate-specific antigen (PSA), Fernandez-Sanchez et al. made use of screen-printed carbon electrodes (SPCEs) coupled together with an LFA. In this case, the SPCE with an interdigitated electrode configuration was turned upside down and positioned over the Ab capture line of the LFS. Here, they used urease as a signal tracer which can catalyze hydrolysis of urea substrate into ammonia. The increase of pH due to ammonia thus resulted in degradation of the polymer layer coated on the electrode in which a change in the capacitance was monitored [83]. It should be noted that even though the authors achieved a satisfying detection limit for determination of free and total PSA levels, the response time was relatively long especially at low PSA concentrations (> 700 s). This could be attributed to the complex signal attainment, i.e., hydrolysis to generate an intermediate to subsequently trigger polymer degradation, which is highly dependent on diffusion of urea and ammonia. The poor contact between electrodes and the Ab capture line could result in low sensitivity, long response time, and poor reproducibility of the assay.

In a similar integration approached, Lu et al. combined SPE with an immunochromatographic strip and used it together with a coupling strategy of bismuth ions to the antibody. Here, the electrodes were placed underneath the strip directly at the test zone. The bismuth ions were released by addition of 1 M HCl and then detected via square wave anodic stripping voltammetry (SWASV) in order to quantify the target analyte [76]. Furthermore, QDs were also implemented in such types of LFS, where the Cd present in the QD was also detected via SWASV. This strategy proved to be highly sensitive and an LOD of 20 pg/mL of PSA was achieved for QDs either made from CdS@ZnS or CdSe@ZnS [75, 91]. The stripping analysis-based approach is highly advantageous to the sensitivity of EC-LFA, in particular with an external electrode, as an efficient recruitment of signal tracer to the electrode surface could be achieved. The contact between electrodes and LFS may be less prone to affect the sensitivity and reproducibility in comparison to the work shown by Fernandez-Sanchez et al. [83]. However, when SPCEs are used, pre-treatment is necessary to ensure sufficient hydrophilicity and surface homogeneity as they are of importance in attaining greater electrodeposition of metal ion tracers. Further research attempt in this area should focus on developing environmentally friendly metal nanoparticle tracers, e.g., AuNPs, and stripping analysis without using mercury.

A similar integration system was implemented for detection of alpha-fetoprotein (AFP) in human serum samples. The sandwich-type immunoassay contained HRP and anti-AFP antibody co-immobilized on AuNPs. HRP induces catalytic oxidation of *O*-phenylenediamine (OPD) in the presence of H_2_O_2_ that can be detected by SWV [74]. It should be noted that the electrochemical measurement in a stagnant condition, especially in voltammetry-based techniques, requires a confined detection area. In order to do this, the authors simply created two lines as a hydrophobic barrier on the LFS, where the test line was located in between, by using a liquid blocker pen mini super pap. This technique has been also implemented with stripping analysis after formation of immunocomplex on LFS [75, 91].

Sinawang et al. used screen-printed gold electrodes (SPGEs) to immobilize capture antibodies against NS1 protein directly on the electrodes, thus forming a sandwich immunoassay with the detection antibodies and ferrocene molecules attached to AuNPs. The SPGE was placed at the end of the cellulosic LFS (Fig. 3A (ii)) where the detection was performed by CV [90]. The work highlighted the ability to integrate the modified SPGEs into a compact cassette that further assists a precise alignment of lateral flow on electrode surface as well as a tight contact between electrodes and LFS, thus minimizing variation between devices.

In addition to placing a whole electrode piece on the top, at the bottom, and at the end (Fig. 3A, B), the NC membrane can be sandwiched in between two electrode pieces which serve as a working and a counter/reference electrode as illustrated in Fig. 3C [79, 94]. This electrode assembling configuration is highly attractive for implementation with reversible redox markers as the distance between the two electrodes defined by the thickness of thin-layer paper could be sufficiently small for redox cycling [95].

**Fig. 4:**
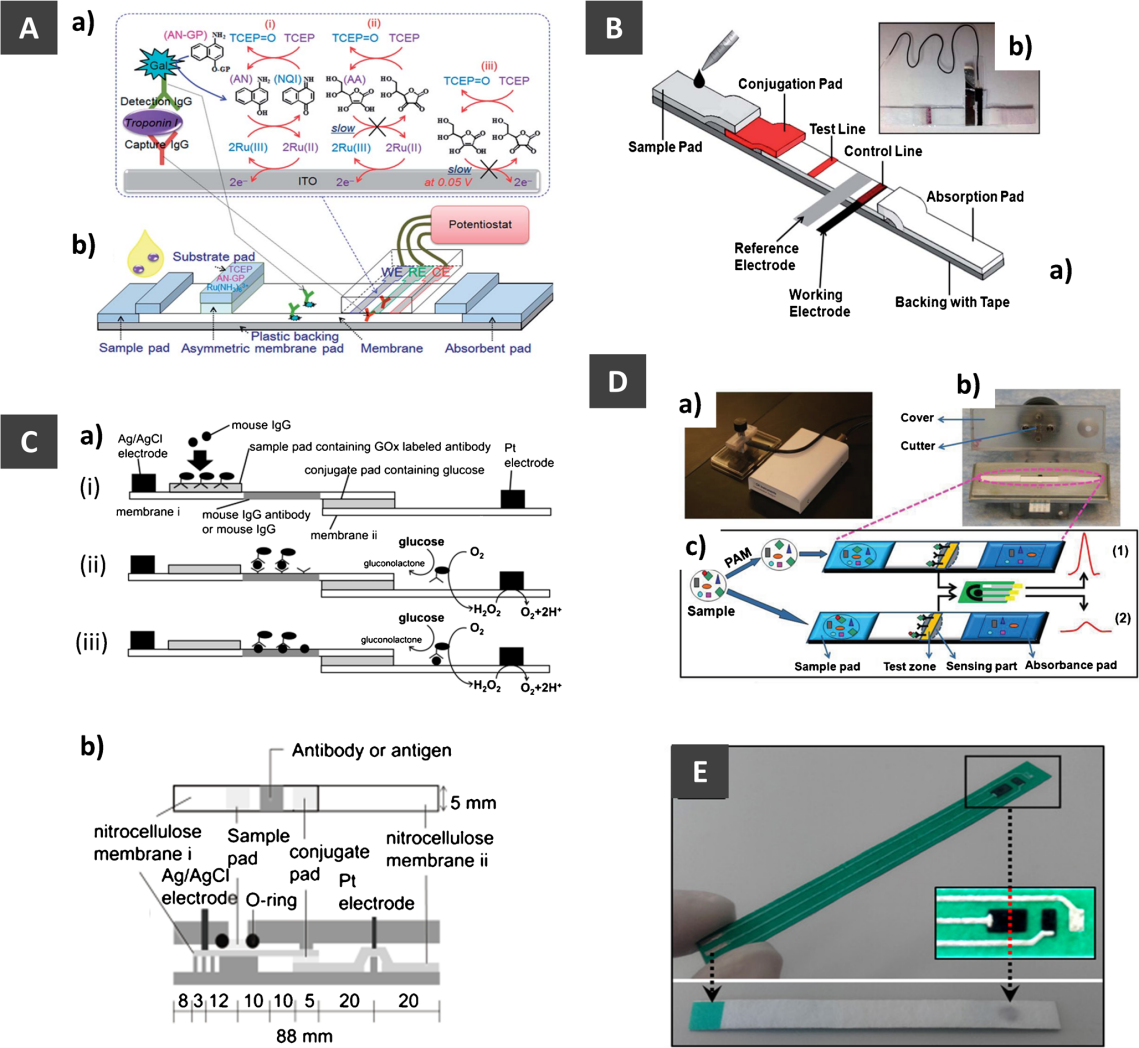
Representative exemplary EC-LFAs with various electrode configuration integrated into LFS as well as assay platforms. **A** ITO electrode-coated glass plates assembled onto LFS (b) and detection principle (a), reprinted from [82] with permission from Royal Society of Chemistry. **B** The CNT conductive paper integrated immunostrip (a) and photograph of actual device adapted from [79] with permission from Royal Society of Chemistry. **C** The single-step quantitative EC-LFS using glucose oxidase (GOx) as signal tracer (a), and components of LFS at the top and side views (b) adapted with permission from [88]. Copyright (2017) Japan Society for Analytical Chemistry. **D** Integrating cutter into an LFS holder for precisely cutting the test line adapted from [73] with permission from American Chemical Society. **E** Internal screen-printed electrodes on LFS reprinted from [80] with permission from John Wiley and Sons

A wide variety of electrodes other than SPEs, e.g., indium tin oxide (ITO) or electrodes made from carbon nanotube (CNT) paper, have been implemented in EC-LFS (Fig. 3E). ITO was coated on a glass substrate as a three-electrode setup via photolithography, placed in contact with the NC membrane and clamped before the absorbent pad where the working electrode (WE) was positioned at the test zone (Fig. 4A) [82]. In addition, electrodes made from Toray paper coated with Pt/C and AuC acting as cathode and anode, respectively, of a microfluidic fuel cell assembling in the EC-sandwiched LFS format (Fig. 3C) could serve as a good candidate for electrochemical sensing in LFAs [94]. A colorimetric and electrochemical detection was combined on an LFS containing a paper electrode for the detection of a DNA oxidative damage biomarker (8-OHdG) (Fig. 4B). A conductive CNT paper strip was used as a WE, being placed on the control line, whereas a copper paper painted with Ag/AgCl ink was implemented as a reference electrode (RE). The detection limits achieved via CA and colorimetric detection were found to be very similar in the range of 5–8 ng/mL [79].

Apart from flat electrodes, Pt wire has been also employed as a WE in an LFS that consisted of two NC membranes set in contact at the conjugate pad (Fig. 4C). In this work, acrylic substrates were used to seal the membranes together with the Pt wire (WE) and Ag wire (RE) with screws. The enzyme-labeling strategy utilized IgG antibody and glucose oxidase (GOx), to detect mouse IgG via CA [88]. This design permits the quantitative electrochemical detection in a single step as all necessary reagents are accommodated within the LFS.

In fact, using external electrodes in LFS could minimize electrode fouling from complex sample matrices because binding between bio-receptors and analytes can take place only on the LFS prior to integrating to an electrode. By far, external electrodes produced by screen-printing technology are more attractive and promise simpler integration into LFS than the others, e.g., CNT paper and Pt wire. The current screen-printing technology commonly provides SPEs with high reproducibility from electrode-to-electrode and batch-to-batch. However, after their integration into LFSs, good reproducibility is often hard to achieve which is mainly attributed to variation of contact strength between electrodes and LFS. This problem has been overcome by using a cassette [90] or lamination [79].

### Ex situ electrochemical detection

A different strategy for electrochemical detection developed in combination with an LFA involves cutting the test line after the assay is performed. For example, in order to detect testosterone, Inoue et al. formed the immunocomplex as normally conducted in LFAs but the test line was later cut and transferred to an external electrochemical cell to determine the activity of the HRP that labeled testosterone Ab. The quantification was performed in ferrocene methanol and H_2_O_2_ via CA [81]. Similarly, Wang et al. developed a system by coupling AuNPs with HRP and antibody against Hg (II) ions. The test zone was cut and added into an electrochemical cell containing the substrate solution. The quantitative detection was achieved by SWV based on the HRP reaction catalyzed by the oxidation of OPD in the presence of H_2_O_2_ [87].

Manually cutting a test zone for further electrochemical measurement may cause variation between assays. This problem could be overcome by assembling LFS in a housing device with an integrated cutter that is located over the test zone as shown by Du et al. (Fig. 4D). Herein, after precise cutting, the test zone was dropped directly into the microelectrochemical cell located underneath [73]. Followed by addition of reaction reagent, e.g., substrate for enzyme, detergent for liposome, or acid solution for QD tracer, to the microelectrochemical cell, the electrochemical measurements can be performed in a more reproducible manner.

To achieve the measurement by affordable home-based electrochemical devices such as a personal glucose meter (PGM), some studies have demonstrated assay platforms that can produce glucose as a signal generation molecule. For example, Zhu et al. employed invertase/Ab-AuNP as a signal tracer in LFA for a sensitive quantification of 8-OHdG and PSA. The immunocomplex was initially performed on a traditional LFS where the test and control lines were cut and immersed into sucrose solution. The invertase can convert sucrose into glucose which can be subsequently measured by a PGM [86]. The use of PGM was also established for quantification of phosphor-p53^15^ (a biomarker for gamma radiation exposure). Instead of an enzyme, liposomes encapsulating glucose were used as a signal tracer. The cut test line was transferred in a detergent solution to release glucose prior to measuring by PGM [96].

Performing electrochemical detection off-LFS is actually similar to the case of external electrodes. Nevertheless, ex situ electrochemical detection may provide better sensitivity and reproducibility as the available electrode surface is not hindered or interfered by NC membrane laid on top or underneath. Among all examples described above, the signal transduction strategy based on glucose detection is the most attractive manner to implement in real-world applications owing to the well-established technology of PGM. However, it should be noted that all strategies based on ex situ detection still introduce inconvenience to users.

### Internal electrodes

With the progress in screen-printing technology and other electrode fabrication techniques, it becomes feasible to generate electrodes onto or into LFSs. As shown in Fig. 4E, a three-electrode system can be screen-printed directly on the LFS. The fabricated EC-LFA device was applied for the quantification of myeloperoxidase (MPO) in human serum [80]. Here, the sandwiched immunomagnetic complex was initially formed off-strip prior to transferring the solution to LFS for separation purpose. The complex was trapped over the electrode by a magnet placed underneath. Nevertheless, the proposed work encountered some shortcomings with respect to the retention of immunomagnetic complex over the specified electrode area during washing on LFS. Here, while large solution volume assists the flow, the confinement of the immunomagnetic complex was troublesome. Additionally, employing a large and strong magnet could induce clustered immunomagnetic complexes and interfere the subsequent electrochemical signal.

Human cardiac troponin T was also detected using an internal SPE underlying a lateral flow membrane. Unlike the previous study [80], the capture antibodies (anti-cardiac troponin T) were modified onto the electrode and the sandwich assay was conducted together with the enzyme HRP-labeled antibodies [84]. With respect to the handling, the strategy is more user-friendly than immunomagnetic separation.

Overall, the strategies mentioned above possess their own pros and cons. Placing commercially available SPE or other electrodes above, underneath, or at the end of an LFS is a straightforward manner which combines existing LFA technology with a well-established and reliable electrochemical transducer. However, intimate contact between electrode surface and LFS has to be established to ensure the desired detection sensitivity. Moreover, too tight or too loose contact may lead to poor reproducibility between LFS devices. Therefore, chip holders that can specify the exact location and provide consistent contact between LFS and electrode should be developed in this case. Similar to on-LFS detection, the strategy based on cutting the test line and performing electrochemical measurement ex situ benefits from the well-established technologies that render reliable results of the measurement. In addition, neither intimate contact nor specific electrode location has to be concerned. However, the strategy may suffer from poor reproducibility due to improper handling by end-users. A cutter built into the LFS holder demonstrated by Du et al. could be a good solution [73]. However, creating electrodes directly on the LFSs is more attractive than the aforementioned strategies. Despite holding a great potential to enable robust EC-LFA, LFAs with internal electrodes have been so far scarcely explored which requires more research attention in the future.

## Outlook

A lot of effort focused on the improvement of the sensitivity of LFAs over the last decades, yet it is still a long way to move this technology towards truly challenging applications. This includes not surprisingly the need to detect a variety of target analytes at low concentrations in a rapid manner while maintaining the key platform features of portability, low cost, and easy adaptation to use in areas with limited resources. In our opinion, the integration of electrochemical detection into a LFA is highly desirable, enabling quantitative detection with high sensitivity for precise diagnostics that should come into focus in the future. Missing so far are studies to better understand the influence of flow rates on the electrochemical performance and how this can be engineered to favor a better performance. Electrochemical transducers have the potential to easily be further extended to multiplex testing, for example, using nanoparticles made from different encoding metals that can be detected simultaneously by stripping voltammetry on a single electrode. Furthermore, their simple fabrication can be easily extended to array production, allowing modifications with different immobilized receptors for a broad range of analytes. Potential interferences from sample matrices can be avoided by dilution of the sample or via magnetic separation. The former strategy obviously requires high-performing electrochemical transducers. The latter could be beneficial in case the electrochemical transducer is not sensitive enough, as the use of magnetic beads allows the complex to be entrapped on the electrodes, promoting the tracer in close contact to the electrode surface.

In addition, current EC-LFAs rely mostly on well-established commercially available electrodes such as SPEs. The proposed integration strategies into LFS are however not yet robust as they require some extra efforts in terms of instrumentation and human inputs. It would be therefore more favorable to develop LFS with self-contained internal electrochemical transducers, but this has been rarely demonstrated so far. The development would make EC-LFAs highly competitive to widely used optically based detection but with rather relatively low cost and high sensitivity. In order to robustly integrate electrodes into LFSs, screen-printing electrode materials onto nitrocellulose membrane could be a promising strategy. However, the limited active surface area may result in poor assay sensitivity. Therefore, it is much more desirable to generate electrodes with an elevated electroactive surface area for EC-LFA.

Furthermore, electrochemical transducers allow their versatile tuning by modification with nanomaterials. Nanomaterials like nanofibers can easily be modified to bear different functionalities not only as part of the transducer but also for sample preparation, such as trapping interfering molecules prior to reaching the transducers.

Considering that strategies for sensitive and fast detection are available, have been well-studied. and rather well-understood as described in this article, efforts should now be oriented towards innovation needed for their commercialization as POC devices. The simplicity of the assay itself will need much attention as successful EC-LFAs must avoid additional steps. Inherently, electrochemical transducers benefit from inexpensive fabrication techniques, important for single-use devices. Sustainable production and disposal must be studied and may be not too challenging for carbon-based transducer materials.

Nowadays, it is obvious how bulky, expensive lab equipment can be replaced by small, handheld, and useful gadgets. An important example is the potentiostat, which was miniaturized and can now easily be plugged into a smartphone and used for measurement. Unique features make the implementation of high-performance sensors and thus adaptable to everyday life. With a simple Bluetooth connection, the potentiostat can be connected to the smartphone, which already accounts for high computing power similar with bench computers. This enables not only the possibility of home testing but also an easy display of the results, which can be automatically saved into a shared cloud or transferred immediately to doctors or data scientists. Additionally, in a suited application software machine learning can be encoded, which can make data analysis more accurate and can easily identify possible problems. Considering the great impact electrochemical transducers had on the point-of-care market such as in the glucose sensors, EC-LFAs will be able to harness all of the associated advancements and thus significantly improve the traditional lateral flow assay and hence further their success in point-of-care applications.
